# Evaluation of fibromyalgia frequency and quality of life in Notalgia paresthetica patients

**DOI:** 10.1007/s00403-024-03027-8

**Published:** 2024-06-01

**Authors:** Nihal Sarı, Sevgi Kulaklı, Işıl Deniz Oğuz, Burak Akşan, İlker Fatih Sarı

**Affiliations:** 1 Department of Dermatology and Venereology, Bulancak State Hospital, Giresun, Turkey; 2 Department of Dermatology and Venereology, Faculty of Medicine, Giresun University, Giresun, Turkey; 3 The Department of Physical Medicine and Rehabilitation, Faculty of Medicine, Giresun University, Giresun, Turkey

**Keywords:** Fibromyalgia, Neuropathic pruritus, Notalgia parestetica, Pain, Quality of life

## Abstract

Based on the presence of chronic pain and the potential use of common treatment agents in Notalgia Paresthetica (NP) and Fibromyalgia Syndrome (FMS) for improvement, we aimed to investigate the frequency of FMS symptoms in NP patients and its impact on quality of life. This study is a case control cohort study including 26 patients diagnosed with NP and a total of 26 controls matched for age and gender. The 2016 revised fibromyalgia diagnostic criteria by the American College of Rheumatology (ACR) were used to inquire about FMS diagnosis criteria in the study. According to the 2016 ACR revised FMS diagnostic criteria, the frequency of FMS was significantly higher in the patient group (n = 9, 34.6%) compared to the control group (n = 2, 7.7%) (p = 0.042). The Wide Pain Index (WPI) score in the control group was 2.00 (3.25), while in the patient group, it was 4.00 (8.00), with a statistically significant difference between them (p < 0.035). Furthermore, significant statistical differences were found between the two groups in terms of Symptom Severity Scale (SSS), Fibromyalgia Score (FS), and FIQ (p < 0.035, p < 0.001, p < 0.001, respectively). In NP patients with accompanying FMS, Dermatology Life Quality Index was significantly more affected compared to those without FMS (p = 0.025). In conclusion, we recommend that NP patients be questioned about FMS, which is characterized by generalized pain, as well as regional neuropathic symptoms. Treatment success can be enhanced by using common agents in the treatment choice for accompanying FMS.

## Introductıon

**Fig. 1:**
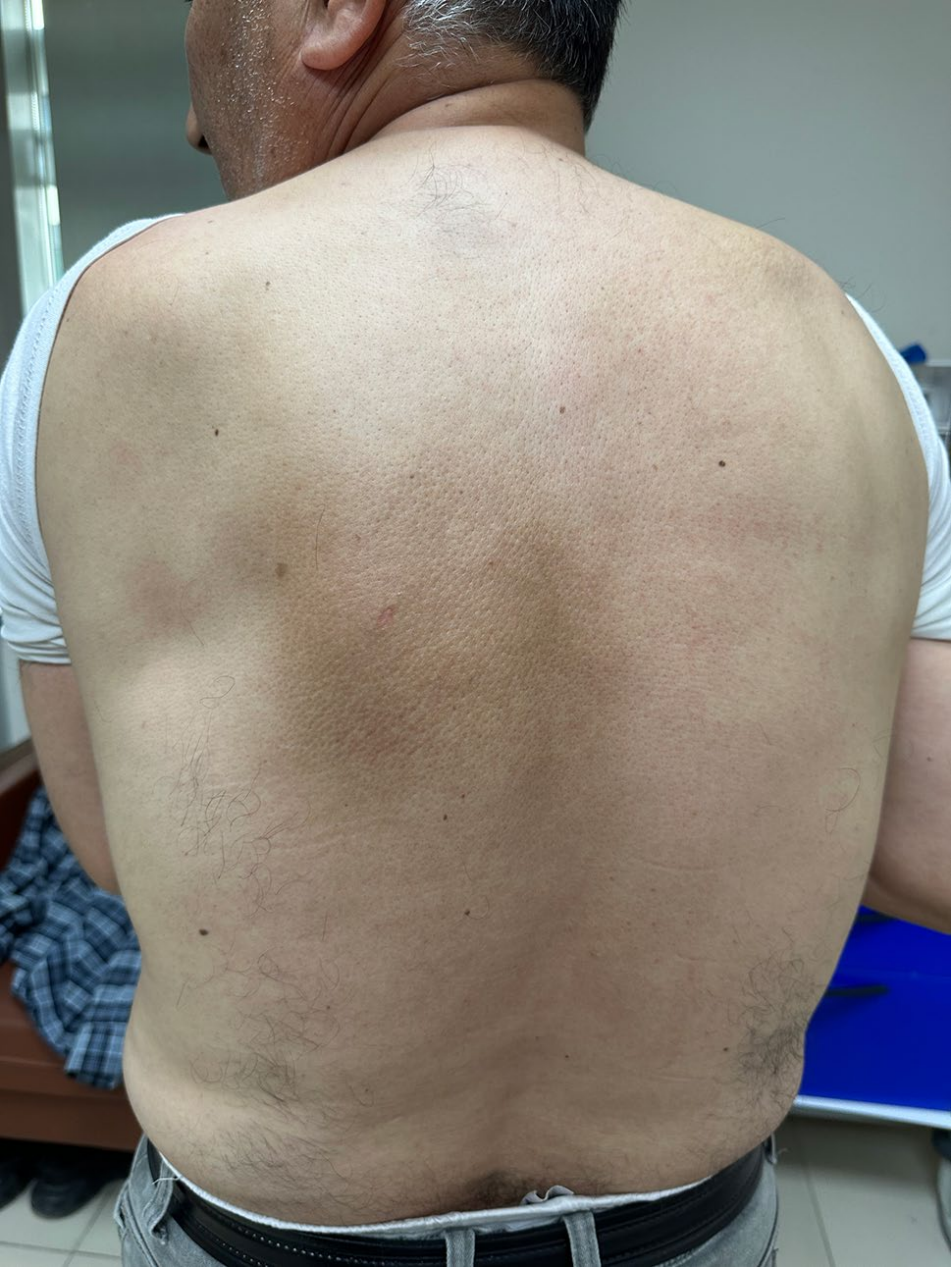
Unilateral hyperpigmented brownish patch in the infrascapular area in male patient

Notalgia Paresthetica (NP) is a sensory neuropathy characterized by well-defined hyperpigmented macules and patches located medial and inferior to the scapula [1] (Fig. 1). It is often accompanied by various sensory symptoms such as itching, painful burning, paresthesia, dysesthesia, or hyperesthesia. Although its etiopathogenesis is not fully understood, factors such as genetic predisposition, increased dermal innervation, and viscerocutaneous reflex mechanisms are emphasized [2]. In addition, the changes that cause the disease can often be attributed to radiculopathy in the primary dorsal branches of the spinal nerves. A study detecting vertebral column disease in approximately 70% of NP patients also supports this theory [3].

Fibromyalgia Syndrome (FMS) is a common chronic pain condition in the community. It may be accompanied by various symptoms such as body aches, tenderness, fatigue, anxiety, cognitive, mood, and sleep disorders. According to the 1990 American College of Rheumatology (ACR) criteria, the average prevalence of FMS diagnosed patients worldwide is approximately 2.7% [4]. While this rate is as low as 0.4% in Greece among countries, studies have found a prevalence as high as 8.8% in our country [5, 6]. It is thought that increased pain pathways in the disease develop on the basis of central sensitization [7].

Considering that both NP and FMS feature chronic pain as the main symptom and common treatment agents are used in their treatment, there may be a correlation between the two diseases. Therefore, we aimed to investigate the frequency of FMS symptoms and its impact on quality of life in NP patients.

## Materıals and methods

This study was conducted between May 2023 and January 2024 at Giresun University Training and Research Hospital and Giresun Bulancak State Hospital Dermatology outpatient clinics. The study was conducted in accordance with the Helsinki Declaration and was approved by the Giresun Training and Research Hospital Clinical Research Ethics Committee (approval number: 27.04.2023/12, date: 28.04.2023). Written informed consent was obtained from each participant.

This case control cohort study included 26 patients diagnosed with NP and a total of 26 controls matched for age and gender. Patients diagnosed with NP with clinical and/or histopathological findings, aged 18 and over, and without any chronic, neurological, or psychiatric diseases were included in the patient group. Patients under 18 years of age, pregnant, and breastfeeding were excluded from the patient group. Individuals over 18 years of age who applied to the dermatology outpatient clinic for nevus monitoring were included in the control group. Individuals with any chronic systemic, neurological, or psychiatric diseases were excluded from the control group.

### Surveys

Demographic data of all patients were collected, and the assessment for the diagnosis of FMS was conducted by an experienced physical medicine and rehabilitation specialist. In this study, the 2016 ACR revised fibromyalgia diagnostic criteria were questioned. The scale used in the ACR diagnosis criteria consists of two parts: Widespread Pain Index (WPI) and Symptom Severity Scale (SSS). WPI indicates the number of out of a total of 19 subregions in 5 main areas (upper right, upper left, lower right, lower left, axial region) where pain has been present in the past week. The total score ranges from 0 to 19. SSS evaluates fatigue, waking up unrested, and cognitive symptoms on a scale of 0–3 points for each existing symptom severity in the past week. Additionally, this scale included questions about abdominal pain and cramps, depression, and headache in the past 6 months (0–1 point). The total score ranges from 0 to 12. According to the 2016 diagnostic criteria, individuals experiencing widespread pain and other symptoms for three months are diagnosed with FMS. Widespread pain is defined as pain present in at least four out of five regions excluding jaw, chest, and abdomen. Additionally, for diagnosis, patients were required to have WPI ≥ 7 and SSS ≥ 5 or WPI 4–6 and SSS ≥ 9 [8].

To measure the clinical severity and functional status of patients diagnosed with FMS, the valid Turkish version of the Fibromyalgia Impact Questionnaire (FIQ) was used. The forms were completed by patients in the outpatient clinic and took approximately 5 min to complete. FIQ forms consist of 10 self-administered scales related to physical functioning, employment status, depression, anxiety, sleep, pain, stiffness, fatigue, and well-being (scores ranging from 0 to 80). The first item consists of 11 questions, each scored from 0 to 3. The second and third items require marking the number of days in the past week for " being affected by the disease " and "not being able to go to work," respectively. The remaining seven questions are related to the severity of symptoms, pain, fatigue, restless sleep, stiffness, anxiety, and depression, and responses are evaluated on a 10-point scale. Higher scores on FIQ indicate higher disease severity [9].

Additionally, the Dermatology Life Quality Index (DLQI) questionnaire was administered to the patient group. This questionnaire reflects changes in quality of life related to symptoms, patients’ feelings, daily activities, methods of assessing leisure time, school/work life, individual relationships, based on treatment. Generally, responding considering patients' conditions in the past week is recommended. The questions have 4 options, and there are a total of 10 questions. Scoring was done as follows: "Very much = 3 points", "A lot = 2 points", "A little = 1 point", "Not at all = 0 points", "Not relevant = 0 points". The total score ranging from 0 to 30 is calculated by summing the responses to each question. Higher scores on DLQI indicate lower quality of life.

### Sample size

G*Power (V3.1) software (Informer Technologies, Inc., Los Angeles, USA) was used to calculate the required sample size[10]. Using data from a previous study, the effect size in our sample size calculation was found to be 0.99. Based on a power of 80% and a 5% level of significance, the total sample size required was calculated as 34.

### Statistical analysis

Statistical analysis was performed using SPSS version 23.0 (IBM Corporation, Armonk, USA). The normal distribution of variables was examined using the Shapiro–Wilk Test. Continuous data with normal distribution were shown as mean ± standard deviation, and continuous data without normal distribution were shown as median (interquartile range). Categorical data were presented as frequency distribution and percentage. Quantitative data between the groups were compared using the independent samples t-test or Mann–Whitney U test according to the normality of the data. Pearson's Chi-square test or Fisher's Exact Test was used for comparing categorical variables. A p-value of < 0.05 was considered statistically significant.

## Results

This study included 26 NP patients and 26 healthy controls. The mean age of the patient group was 52.50 ± 12.71 years, while the mean age of the control group was 52.35 ± 13.99 years. Of the total 26 NP patients, 24 (92.3%) were female and 2 (7.7%) were male, while in the control group, 22 (84.6%) were female and 4 (15.4%) were male. According to the 2016 ACR revised FMS diagnostic criteria, the frequency of FMS was significantly higher in the patient group (n = 9, 34.6%) compared to the control group (n = 2, 7.7%) (p = 0.042). The WPI score in the control group was 2.00 (3.25), while in the patient group, it was 4.00 (8.00), with a statistically significant difference between them (p < 0.035). Furthermore, significant statistical differences were found between the two groups in terms of SSS, FS, and FIQ (p < 0.035, p < 0.001, p < 0.001, respectively) (Table 1). 100% of NP patients diagnosed with FMS were women. When comparing NP patients with and without accompanying FMS, significant statistical differences were found in terms of WPI, SSS, FS, and DLQI (p < 0.001, p = 0.010, p < 0.001, p = 0.025, respectively) (Table 2).

**Table 1:** The presence of fibromyalgia syndrome and assessment of Widespread Pain Index, Symptom Severity Scale, Fibromyalgia Score, and Fibromyalgia Impact Questionnaire score in notalgia paresthetica and control group

	Notalgia paresthetica (n = 26)	Control (n = 26)	P value
Age (years), mean ± SD	52.50 ± 12.71	52.35 ± 13.99	*0.967*
Sex Female Male	24 (92.3%) 2 (7.7%)	22 (84.6%) 4 (15.4%)	0.668^*^
Diagnosed with FMS	9 (34.6%)	2 (7.7%)	***0.042***
WPI, median (IQR)	4.00 (8.00)	2.00 (3.25)	***0.035***
SSS, mean ± SD	7.19 ± 3.37	4.19 ± 2.95	***0.001***
FS, mean ± SD	12.96 ± 6.68	7.27 ± 5.05	***0.001***
FIQ, mean ± SD	54.68 ± 22.06	25.65 ± 15.14	** <*0.001***

Significant P-values are shown in bold italics

*FMS* fibromyalgia syndrome, *WPI* Widespread Pain Index, *SSS* Symptom Severity Scale, *FS* Fibromyalgia Score, *FIQ* Fibromyalgia Impact Questionnaire, *SD* Standard deviation, *IQR* interquartile range

^*^Fisher’s Exact Test

**Table 2:** Comparison of patients with and without fibromyalgia syndrome (FMS) according to the demographics, clinical features, FMS scores and Dermatology Life Quality Index in the patient group with Notalgia Paresthetica

	Notalgia Paresthetica with FMS (n = 9)	Notalgia Paresthetica without FMS (n = 17)	*P value*
Age, years, mean ± SD	50.67 ± 14.56	53.47 ± 11.99	*0.603*
Sex Female, n, (%) Male, n, (%)	9 (100.0%) 0 (0%)	15 (88.2) 2 (11.8)	0.529^*^
Duration of notalgia, months, median (IQR)	60.00 (270.00)	48.00 (90.00)	*0.287*
WPI, median (IQR)	10.00 (8.50)	3 (3.50)	** < *0.001***
SSS, mean ± SD	9.44 ± 2.40	6.00 ± 3.24	***0.010***
FS, mean ± SD	19.67 ± 4.80	9.41 ± 4.42	** < *0.001***
FIQ, mean ± SD	63.24 ± 19.72	50.15 ± 22.42	*0.154*
Localization of lesion, n (%) Unilateral Bilateral	5 (55.6%) 4 (44.4%)	15 (88.2%) 2 (11.8%)	0.138^*^
DLQI, median (IQR)	8.00 (13.00)	3.00 (6.00)	***0.025***

Significant P-values are shown in bold italics

*FMS* fibromyalgia syndrome, *WPI* Widespread Pain Index, *SSS* Symptom Severity Scale, *FS* Fibromyalgia Score, *FIQ* Fibromyalgia Impact Questionnaire, *DLQI* Dermatology Life Quality Index, *SD* Standard deviation, *IQR* interquartile range

^*^ Fisher’s Exact Test

## Dıscussıon

NP was first described by Astwazaturow, a Russian neurologist, in 1934. NP is known as a sensory neuropathy that affects the posterior cutaneous nerves of the upper branches of the T2-T6 spinal nerves [11]. According to a hypothesis, compression of the musculoskeletal system in the posterior branches of the peripheral nerves is the main factor causing sensory neuropathy. The term "Notalgia" in NP is inspired by the Greek words "notos" (back) and "algos" (pain) [12]. FMS is a partially related but completely different entity. FMS is the third most common musculoskeletal disorder in terms of prevalence, after low back pain and osteoarthritis [4]. FMS, one of the main causes of chronic widespread pain, is often accompanied by fatigue, sleep disturbances, and functional symptoms. Both NP and FMS involve pain pathways in their underlying mechanisms, and the use of common treatment agents (such as gabapentin, pregabalin, amitriptyline, etc.) suggests a potential association between them. Based on this hypothesis, we aimed to investigate the frequency of FMS symptoms in NP patients and its impact on quality of life.

Both NP and FMS are diseases whose etiopathogenesis has not yet been fully elucidated. The relationship between FMS and many dermatological diseases has been investigated in previous literature. In a study by Torresani et al., the prevalence of FMS in patients with chronic urticaria was found to be 70.6%, which was statistically significant compared to the control group. They emphasized that this rate was unexpectedly high [13]. Additionally, in a study by Kulaklı et al., the incidence of FMS in patients with chronic urticaria was determined to be 26.4% [14]. The frequency of FMS has also been investigated in another chronic inflammatory skin disease, rosacea. In a study conducted by Acar et al., FMS diagnosis was found in 37% of rosacea patients, which was statistically significant compared to the control group. They attributed this significant association to neurogenic inflammation, which is involved in the pathogenesis of both diseases. FMS is actually a non-inflammatory rheumatologic disease [10]. The prevalence of fibromyalgia has also been found to be high in other chronic inflammatory skin diseases such as acne and Behçet's disease in literature studies [15, 16]. In our study, the frequency of FMS in NP patients was significantly higher compared to the control group (p = 0.042).

NP is a rare condition that concerns both dermatology and physical medicine and rehabilitation fields. Its etiopathogenesis is still under investigation, with characteristic sensory neuropathy thought to arise from degenerative processes in the spine or impegement of spinal nerves secondary to musculoskeletal system pressure. Sometimes, lumbar disc herniation or degenerative disc diseases may also accompany NP [17]. There are no cohort studies containing clear data on its frequency. In our study, the frequency of FMS in NP patients was found to be significantly higher compared to healthy controls, as well as the WPI, SSS, FS, and FIQ. In studies conducted in our Turkish population, the frequency of FMS is normally 8.8%, whereas in our study, the frequency of FMS in the healthy control group was found to be 7.7%, which is similar to the literature [6].

NP is a disease that often requires repeated healthcare facility visits and can be challenging to respond to treatment options. Patients may develop nosophobia due to pigmentation in the lesion area. Burning and pain in the affected area, are also important symptoms of NP, which is cause of peripheral neuropathic itching. The diversity and abundance of pain areas in the responses given to the FIQ in our study strongly indicate a coexistence with FMS, which is characterized by widespread body pain. Additionally, in our study, the FMS impact score of the patient group was found to be significantly higher compared to the score of the control group. Although NP presents with localized regional symptoms and FMS with widespread symptoms, they share similar treatment modalities. Duloxetine and pregabalin are the most effective pharmacological agents in the treatment of FMS. Additionally, amitriptyline is more effective in improving sleep, fatigue, and overall quality of life [18]. When it comes to NP treatment, various methods stand out, including physical modalities (physiotherapy, exercise, TENS), topical agents (capsaicin, tacrolimus, topical anesthetics such as lidocaine/prilocaine), systemic agents (gabapentin, oxcarbazepine, amitriptyline), botox, anesthetic blocks, narrowband UVB, and acupuncture [1]. Especially, gabapentin is prominently used among pharmacological agents in treatment, and the efficacy of amitriptyline and duloxetine has been demonstrated in a limited number of case reports [19, 20]. In our study, we had a patient who underwent almost all treatment modalities. NP, like many treatment-resistant cases, is a disease without mortality but can adversely affect patients' quality of life [21].

Both pain and itching are transmitted to the central nervous system via the same type of myelin-free, slow-conducting "C" fibers. The discovery that chronic pain and chronic itching are sensitive to similar drug treatments such as gabapentin, pregabalin, clonidine, and local anesthetics further underscores their clinical similarities. For example, gabapentin is commonly used to treat neuropathic pain and neuropathic itching alike [22]. Based on this similarity, the itching and burning symptoms in NP have complex mechanisms that can affect each other through common pathways with the pain symptoms in FMS. Indeed, our study similarly found a higher frequency of FMS in NP patients compared to the healthy population.

The prevalence of FMS, especially in the adult population, is 8–9 times higher in women compared to men [23]. In fact, 90% of fibromyalgia patients seeking medical treatment are women [24]. In our study, when we looked at the gender of patients with NP who also had FMS, all of them were female. Although there was no significant difference in terms of gender between the two groups, this high ratio supports the female gender dominance in FMS prevalence.

## Lımıtatıons

Although our study is the first to investigate the relationship between these two diseases, its most significant limitation was the limited number of patients. Scientific publications related to NP mostly consist of case reports and generally involve studies with a limited number of patients. Due to the limited number of patients, no data regarding prevalence could be found in the literature. Additionally, despite being conducted at two centers, another limitation is that patients from the same region were included in the study. The detailed evaluation of whether NP patients were using any common treatment agents that could affect FMS symptoms at the time of inclusion in the study was not performed, which is also a limitation.

## Conclusıon

In conclusion, we suggest that patients with NP should be evaluated not only for regional neuropathic symptoms but also for FMS characterized by generalized pain. In cases where FMS coexists, treatment success will be enhanced by using common agents in treatment selection. We believe that our study would benefit from being supported by literature with a larger number of cases.

## Data Availability

The datasets generated during and/or analyzed during the study are available upon reasonable request.
